# Breastfeeding during the COVID-19 pandemic

**DOI:** 10.3389/fped.2023.1120763

**Published:** 2023-06-05

**Authors:** Bwalya Mpelwa Chanda, Xiao-Qing Chen

**Affiliations:** ^1^ Department of Pediatrics, The First Affiliated Hospital of Nanjing Medical University, Nanjing, China; ^2^ Department of Pediatrics, First Affiliation Hospital of Nanjing Medical University, Nanjing, China

**Keywords:** COVID-19, breastfeeding, vertical transmission, characteristics of SARS-CoV-2, COVID-19 vaccine

## Abstract

The coronavirus disease 2019 (COVID-19) caused by severe acute respiratory syndrome coronavirus 2 (SARS-CoV-2) has caused many significant changes to all aspects of day to day life. The disease has spread and reached pandemic proportions. The principle route of transmission is the respiratory route. Infants, pregnant women and breastfeeding mothers have all been affected. Many interventions and guidelines from important societies have been instituted in order to curb the transmission of the disease. These have involved both pharmacological and non-pharmacological methods. COVID-19 vaccines have also emerged as important methods of primary prevention of the disease. But several questions have been raised concerning the safety and efficacy of their use in pregnant and breastfeeding mothers. It has also not been clear if the vaccines are effective in generating a robust immune response in the pregnant women and breastfeeding mothers to confer passive immunity to the fetuses and infants, respectively. And they have not been tested in infants. The aspect of infant feeding has equally been affected. Although breast milk has not been known to serve as the vehicle of transmission of the virus, there is still some lack of uniformity of practice regarding breastfeeding when a mother has SARS-CoV-2 infection. This has led to infant feeding being done by the use of commercial formula feeds, pasteurized human donor breast milk, feeding on the mother's own expressed breast milk by a care giver and directly breastfeeding with skin to skin contact. This is despite breast milk being the most physiologically appropriate type of feed for infants. Therefore the pertinent question remains; should breastfeeding continue during the pandemic continue? This review also seeks to analyse the vast amount of scientific information regarding the subject and to synthesize science-based information

## 1. Introduction

Coronavirus disease 2019 (COVID-19) is a novel type of highly contagious infectious disease caused by a novel type of coronavirus now termed Severe Acute Respiratory Syndrome Coronavirus 2 (SARS-CoV-2). By using RNA (Ribonucleic Acid) sequencing the causative virus was found to be a previously unknown betacoronavirus (52). Because it was previously unknown, the World Health Organization (WHO) named the virus as the “novel coronavirus 2019” (2019-nCoV), and later the disease was renamed as “coronavirus disease 2019” (COVID-19) (112, 113). Coronaviruses are characterized by frequently crossing the species barrier. These events occur in circumstances that are yet to be fully understood; and can occur from animals to humans (zoonoses), or from humans to animals (reverse zoonoses) (6). The disease is believed to have originated from bats (99–107), spilling over to the human beings in which species the infection has continued, even to reach pandemic proportions. And this was not the first occurrence of such a crossing of the species barrier for beta-coronaviruses, as the phenomenon has occurred 3 times in the last 20 years leading to 3 outbreaks of zoonotic type, these being the following; SARS-CoV, MERSCoV, and SARS-CoV-2 outbreaks of 2002/3, 2012 and 2019 to date, respectively (25). Ellis et al. (29) reported that jumping the species barrier is made possible because the genomes of coronaviruses often maintain characteristic features of their wild type as well as including new characteristics.

**Table 1:** Showing the evolution of COVID-19 guidelines.

Date	Guideline	Source of guideline
12th January 2020	Keep COVID-19 patients isolated until evidence of clearance of infection; clinical recovery plus negative RT-PCR on two separate occasions taken ≥24 h apart	(99–107)
18th February 2020	Temporary separation of infants born to SARS-CoV-2 positive mothers	CDC (40)
13th March 2020	Keep mothers with suspected or confirmed SARS-CoV-2 and their new born infants together.	WHO (World Health Organization) (81)
	Mothers should breastfeed normally with direct skin-to-skin contact, kangaroo mother care and rooming in all both during the day and at nigh	
	Mothers to wash hands frequently wear a face mask and to routinely disinfect surfaces.	
4th April 2020	Keep mother and the new born infant together	CDC (40)
	Encourage breastfeeding	
20th May 2020`	Temporary separation of infants born to SARS-CoV-2 positive mothers and	(40)
	Discourage breastfeeding	
27th May 2020	Discharge symptomatic patients 10 days after they who become symptom-free	(99–107)
	Discharge asymptomatic patients 10 days from the day of the positive test	
3rd August 2020	Keep mother and new born infant together	CDC (40)
	Encourage rooming	
	Encourage breastfeeding while observing COVID prevention measures	
27th December 2021	Isolate for 5 days if COVID-19 positive	CDC. (10, 11).
	If asymptomatic or if symptoms are resolving (afebrile for 24 h), isolate for 5 days, and then wear a mask for 5 days when around other people. If febrile, continue to stay at home until fever resolves.	
	If exposed to COVID-19 and unvaccinated or are more than 6 months out from the second mRNA dose and not yet boosted:	
	-Quarantine for 5 days, strict mask use for 5 additional days	
	-OR strict use of face mask for 10 days when around people (when quarantine is not possible).	
	No need to quarantine if boosted and exposed to COVID-19. Only strict use of face mask for 10 days after exposure is recommended.	
	For all exposed, test for COVID-19 at day 5 after exposure; if symptoms occur, quarantine until after a negative COVID-19 test confirms the symptoms are not due to COVID-19	
7th Jan 2022	Irrespective of vaccination status, if COVID-19 positive isolate at home for 5 days. End isolation after 5 days if symptoms are improving and afebrile for not less than 24 h.	ECDC (28)
	Quarantine at home for 5 days if not up to date with COVID-19 vaccine and get exposed to COVID-19. Use face mask for 5 days thereafter.	
	No need to quarantine if up to date with COVID-19 vaccination and exposed to COVID-19 or if a positive COVID-19 test in the past 90 days is confirmed. Get COVID-19 test for 5 days after exposure and use face mask for 10 days after exposure.	
7th July 2022	Close contacts of COVID-19 cases if vaccinated should immediately take COVID-19 test and quarantine for 10 days or quarantine for 14 days if not tested on day 10.	NPHIC. (63)
	Close contacts of COVID-19 cases if vaccinated should immediately take COVID-19 test and quarantine until tests negative for COVID-19. Take COVID test 2–4 days after the first negative test. And practice self-monitoring, use of face masks, avoiding contact with vulnerable populations and maintain distance from other people.	
	Unvaccinated COVID-19 cases should EITHER:	
	-isolate until 24 h after resolution of fever and clinical symptoms improve. And isolate for 10 days after symptom-onset,	
	-OR isolate until 24 h after resolution of fever and improved clinical symptoms. And should have two consecutive negative COVID-19 tests taken at least 24 h apart.	
	Vaccinated COVID-19 cases should EITHER:	
	-isolate until 24 h after resolution of fever and improved clinical symptoms and 6 days onset of symptoms with negative tests on day 6,	
	-OR isolate until 24 h after resolution of fever and improved clinical symptoms. And should have two consecutive negative COVID-19 tests at least 24 h apart.	
21st March 2023	If COVID-19 positive irrespective of vaccination status, stay home for at least 5 days and isolate from others in your home.	CDC. (15)
	End isolation after 5 days if asymptomatic	
	If moderate illness (shortness of breath, difficulty breathing), severe illness (requiring hospitalization) or have a weakened immune system isolate for 10 days	
	If severe disease, consult a doctor before ending isolation, negative test may be necessary.	

## 2. Methodology

Finding reliable and authentic sources of information for citation on the topic of “breastfeeding during the COVID-19 pandemic” was a challenge for the researchers. The researchers used electronic databases using the key word searching methods to locate sources of information on the topic. A methodical search was performed in many databases, among them the Lancet, ABM, ACOG, CDC, WHO, RCOG, New England Journal of Medicine, Google Scholar, Cochrane, UpToDate, from March 2015 up to and including April, 2023. The descriptors used to perform the searches were breastfeeding, breast milk, COVID-19, Coronavirus disease 2019, SARS-CoV-2 vertical transmission, mother-to-child transmission, intrauterine transmission, in-utero transmission, immunoglobulin, COVID-19 vaccine, COVID-19 guidelines. No language restrictions were applied. Full-text articles were identified from online sources.

The credibility of each source was checked one by one so as to avoid citing false, unverifiable and unscientific information. To check for credibility of the information, the researchers found background information on each one of the authors. The researchers had the responsibility of determining that the information cited in the review was credible and valid. Therefore, a list of evaluating criteria consisting of the following was used:

i. Is this the true author of the information?
ii. Is this an authentic and reliable website?
iii. Is the information verifiable and true?
iv. Is this information from original research?
v. Are credible researchers cited by the source?
vi. Is the information concise, logical and well-organized?

### 2.1. Transmission of SARS-CoV-2 to the general population

From the Chinese City of Wuhan the disease spread rapidly across the whole of China, and to the rest of the world (72) leading to, on 30th January, its being declared a Public Health Emergency of International Concern, PHEIC (99–107); and as a global pandemic on March 11th, 2020, by the WHO (99–107). The pandemic continues to cause global disruption and affecting all areas of people's lives, apart from just health. It also affects other facets of human life such as the social, economic, cultural, and political and economic aspects of living globally (99–107). Therefore, there has been particular interest in the disease leading to increased publishing on the disease lately. However, some information is deficient in evidence that originates from science, which could be harmful instead of being beneficial. The need for science-based practices is very important with regard to breastfeeding in women who are suspected or confirmed to be infected with the disease (56).

In the early stages of the pandemic, it was thought that all of the patients that suffered from COVID-19 had been to the seafood market at Hunan in Wuhan. It was also thought that if they had not visited the market, then they must have consumed meat from that had originated from there. But additional investigations revealed that some of them had neither visited the food market nor consumed meat and meat product from there. Thus, the spread of the virus via aerosols or droplets was accepted (55).

The respiratory route is the principal route of transmission for SARS-COV-2, a highly contagious virus (78, 116). Spread also occurs by both direct and indirect contact between people within a distance of 2 m. But transmission over longer distances is unlikely (78). Additionally, Anastassopoulou et al. (6) reported that respiratory secretions are not only expelled during sneezing or coughing, but also during talking, singing, and laughing. The droplets so released are then inhaled. This has since formed the scientific basis for the public health advice of social distancing.

This review, meant for health care professionals, is compiled from the latest available information about Coronavirus Disease-2019 and is not intended to substitute either clinical judgement or expert opinion. It should also be noted that because of on-going research about COVID-19, new information is becoming rapidly available, and suggestions may change as such.

### 2.2. Characteristics of SARS-CoV-2

The SARS-CoV-2 virus is an enveloped positive sense single stranded RNA beta-coronavirus virus ranging from 27 to 33 kb in size and has a crown-like appearance from which it gets the name “corona”. Investigating the virus proved that the organization of its genome is similar to that of the other betacoronaviruses (115, 4). Its genomic sequence is 79% identical to SARS-CoV and 50% identical to Middle East Respiratory Syndrome Coronavirus (MERS-CoV), 88% identical to bat-SLCoVZC45 and bat-SL-CoVZXC21. It was also found to be 96.2% identical to CoV RaTG13 which is a bat CoV (115, 76); with structural genes whose amino acids are over 90% identical to those of SARS-CoV-2. It was also discovered that beyond the similarities in the genome, the proteins that code for genes were also similar; exhibiting 79.5% similarity to SARS-CoV-2 and 51% similarity to MERS-CoV (44, 45, 55). The SARS-CoV-2 genome contains several reading open reading frames (ORFs) whose function is to serve as a template for the production of subgenomic mRNAs and accessory proteins. It also contains putative ORFs interposed between structural genes and code for structural and accessory proteins (82, 59).

## 3. Breast milk dilemma during the pandemic

Even as information regarding the subject is becoming more available, we can make use of what is currently known about breastfeeding and previous outbreaks of infectious diseases to avert the deleterious effects of the COVID-19 pandemic. Additionally, we can refer to current literature in order to inform our position. Interest has also arisen regarding vaccinating the pregnant and lactating mothers against COVID-19.

Lactating women who had been recently diagnosed with COVID-19 were enrolled into a study by Pace et al. (65) in which samples of their breast milk were investigated for SARS-CoV-2 RNA, specific (Immunoglobulin A) IgA and (Immunoglobulin G) IgG. SARS-CoV-2 RNA was not found in any of the 37 breast milk samples. But they contained specific IgA and IgG, and anti-RBD IgA (anti-Receptor Binding Protein IgA). The SARS-CoV-2 Receptor Binding Domain (RBD) has the function of attaching to the Angiotensin Converting Enzyme (ACE-2) receptor before the virus can infect the cell. So, preventing this step from happening by the activity of the anti-RBD IgA may offer protection against infection. Thus, breast milk has potentially protective properties against SARS-CoV-2 to the breastfed infant (65, 16, 4, 48). In a cohort study 285 samples of breast milk collected from 110 lactating mothers were analysed. 65 (59%) of the mothers had tested positive for SARS-COV-2 by nasal or nasopharyngeal swab SARS COV-2 RT-PCR (Reverse Transcriptase Polymerase Chain Reaction) test. 9 (8%) of the mothers were symptomatic but had negative diagnostic tests and 36 (33%) were symptomatic and were not tested. Notably infectious virus was not found in any of the cultures nor was any subgenomic viral RNA a putative marker for infectivity found (47). Another study (71) enrolled 50 pregnant women all of whom were SARS-COV-2 positive. Only expressed breast milk samples from 49 mothers were analysed as one neonate who was delivered by caesarean section at term demised from severe birth asphyxia 4 days after birth. By the 4th day of the peurperium, 1 sample was collected from each mother and all of the samples tested negative for SARS COV-2 by RT PCR. At 1 day of age all the neonates were screened for SARS-CoV-2 by nasopharyngeal swab RT-PCR test. 2 (4%) of the 51 neonates tested positive during which time they were fed on formula. They remained without exhibiting any symptom of the disease. All the breast milk samples were negative for the virus. Thus breast milk is an unlikely vehicle for transmission (71). And as yet, Mother to Child Transmission (MTCT) of COVID-19 through breast milk and breastfeeding has not been known (112, 113).

### 3.1. Potential risk of transmission during breastfeeding, overcame by benefits

Breast milk is the most suitable and ecological feed for the infant (44, 45, 114). Breastfeeding is an important step in mother-infant interaction, and is critical in neonatal early prognosis and growth. Breast milk is indispensable because it is sufficient to nourish and meet the energy needs of children entirely during the first six months of life, with its sufficiency gradually reducing thereafter to not less than half in the next six months (i.e., up to one year), and to a third up to two years of age. Thus, it is the best source of nutrition for infants (108, 109, 110, 111, 5, 84) and should therefore be continued until the child is at least 2 years old (58, 108, 109, 110, 111). Breastfeeding also protects the neonates, infants and children against morbidity and mortality (86). Breast milk also improves survival as it confers passive immunity to the infant as it is rich in immunoglobulins which are very effective against bacterial and viral infections as well as some cancers (23, 19, 84, 67. Breast milk also plays a critical role in the development of a healthy and balanced microbiome (44, 45). Initially there was reasonable concern about the possibility of transmission COVID-19 through breastfeeding because of viral RNA and other viral particles that can be found in breast milk (60, 118). In a study of 140 breastfeeding mothers, SARS-CoV-2 RNA was isolated in 20 (14.29%) of them (68, 35). Also, literature (68) reports that the incidence of vertical transmission is not non-existent but was reported to be about 4.2% when over 800 neonates were reviewed. Another study (79) of 42 asymptomatic SARS-CoV-2 positive pregnant women and their new born infants when they were born reported that 5 out of the 42 neonates (about 11.9%) had strong evidence for possible MTCT of SARS-CoV-2. This study used the WHO criteria for classification and timing of mother-to-child transmission of SARS-CoV-2. Therefore, the risk of vertical transmission should not be overlooked. There is also lacking strong scientific evidence to the effect that there is transmission of the virus through breast milk. This has been proven because the viral materials that can be found in the breast milk of mothers with SARS-CoV-2 are unlikely to be infectious. But breast milk is a likely lasting source of the anti-Receptor Binding Domain (anti-RBD) IgA thereby conferring passive immunity against COVID-19 (4, 60, 118). Breast milk also contains lactoferrin a cationic iron-binding glycoprotein present in mucosal secretions that posses broad-spectrum activity against both DNA and RNA viruses. Both in vivo and in silico studies have shown evidence of lactoferrin activity against SARS-CoV-2 (119, 120). Therefore, important societies like the WHO, UNICEF, and Centers for Disease Control and Prevention (CDC) endorse breastfeeding even during the COVID-19 pandemic albeit with caution that preventive measures should be adhered to. Additionally, they encourage that when it is feasible, the kangaroo mother care should also be practiced (117, 96, 10, 11, 64, 60). And it is important to note that children are less likely to be infected by the disease. This has been linked to the fact that children have low expression of the main receptor for the virus, the ACE-2 receptors (39, 17). And infants have a higher chance of recovery than adults. This is due to the excellent capacity of the epithelial cells of the lungs in children to regenerate, as well as, them not having risk factors like smoking and less physical activity (54, 74).

### 3.2. Breastfeeding practice and promotion in nursery and at home

The critical role that breastfeeding plays is beyond providing nutrition but also conferring passive immunity to the infant, therefore, breastfed infants are less prone to infectious diseases like diarrhoea and when they do suffer from these diseases they have better prognosis (87, 75, 64). Therefore, mothers and their families should be provided with appropriate information regarding safe breastfeeding during the pandemic (84). The mothers should also be taught the “Ten Steps to Successful Breastfeeding” as part of the Baby-Friendly Hospital Initiative (BFHI) which is supported being important societies like the CDC (84). Initiation of breastfeeding should still happen within the 1st hour of birth even during the pandemic (67).

Globally, the percentages of infants being breastfed still need to be improved upon. According to the WHO between 2013 and 2018 only 43% of new born infants initiate breastfeeding within one hour of birth. Only 41% of infants under the age of six months are breastfed exclusively. And only 45% of children are breastfed at the time they are two years of age (87, 98). Due to the many benefits of breastfeeding, the practice should be encouraged and promoted among all lactating women, as well as among expectant mothers. The recommendations of the WHO and UNICEF (42), are that;

i. Breastfeeding should be initiated not more than 1 h after birth;
ii. The infant should be breastfed exclusively until 6 months of age;
iii. Beginning at 6 months of age the infant should be introduced to other foods that are safe and adequate in nutrition while breastfeeding should be continued for not less than 2 years

The above recommendations need to be emphasized because of some of the reported effects of the pandemic on lactation support and breastfeeding rates. These include keeping the mother-infant dyad separated from the rest of the family and other social support networks which reduces available lactation support. Accelerated post-delivery discharges, coupled with reduction of in-person lactation support may also lead to mothers not achieving their breastfeeding targets (24). It has been reported by another study (83) that the first time mothers were the most affected especially during the hours and days after delivery when breastfeeding had not yet been established.

### 3.3. Breastfeeding promotion in neonatal intensive care unit (NICU)

The importance of breastfeeding is even higher for neonates born preterm or premature. Despite that, the rates of breastfeeding are even lower in the Neonatal Intensive Care Unit (NICU) setting. Owing to the many benefits of breastfeeding to the premature neonates, breastfeeding should be encouraged in the NICU. Breastfeeding in the Neonatal Intensive Care Unit (NICU) can be promoted by among others the following ways (33, 57, 36);

i. Family-centred approach
ii. Skin-to-skin contact –kangaroo mother care
iii. Early and frequently expressing breast milk from both breasts; and
iv. Support from peers
v. Lactation counselling
vi. Involving mothers in the care of neonate admitted to the NICU
vii. Use of human donor breast milk

In the event that the milk from the mother is unavailable, human donor breast milk from milk banks can be used. Because it is pasteurized, human donor breast milk has reduced bioavailability than mother's breast milk and may therefore be used only until the mother's breast milk is available (1, 66). Despite a reduction in bioavailability, human donor breast milk is befitting substitute as it effectively inactivates the SARS-CoV-2 virus (95, 85, 64).

### 3.4. Breastfeeding when mother is affected

When a mother has COVID-19 she should breastfeed while adhering to COVID-19 guidelines. She can also express her own breast milk and safely feed it to the infant herself (84). But in the event that a mother is unable to breastfeed because of being very ill, she should be supported by a healthy caregiver by for example, feeding the infant with milk that has been expressed from the mother's breast. If this is not feasible, human donor breast milk sourced from milk banks can also be used. Even in such situations, safety precautions aimed at reducing the risk of transmission of the virus should be followed, among them (93, 64, 10, 11, 69, 60);

i. The caregiver should be healthy and without COVID-19, not exhibiting any of the symptoms of the disease, and preferably vaccinated.
ii. A clean breast pump (preferably a dedicated breast pump), if possible should be used.
iii. The caregiver should wash hands using soap and water prior to touching any pump, bottle parts, or the mother’s breast.
iv. The mother’s breast should also be washed before and after expressing the milk.
v. Wear a mask while administering the breast milk and whenever within 2 m of the baby.
vi. Wear a mask during expression.
vii. Proper pump cleaning after use.

Breastfeeding should be initiated and conducted normally with rooming in starting within the 1st hour of birth. The appropriate breastfeeding technique which includes mother holding the baby skin-to-skin should be encouraged. Further, uninterrupted exclusive breastfeeding for the first six months is encouraged. Thereafter breastfeeding should continue up to the age of two years or beyond, alongside other feeds (67).

## 4. COVID-19 in the neonate

Dhir et al. (26) reported that fewer children than adults have been infected by the disease confirmed by RT-PCR, and that they have excellent prognosis. The infection in neonates most often sets in in the postpartum period, with 2/3 being symptomatic. Because most of the neonates are reported to be well prior to the onset of symptoms of the disease implies that the symptoms are not due to non-COVID causes such as prematurity but are due to the infection itself. The occurrence of the disease in neonates is very infrequent.

But regarding severity of the disease in this age group, Dhir et al. (26) reported that it is more severe in neonates than in older children because when affected the former are more likely to require NICU admission than the latter. And according to Leung (51) neonates run a more severe clinical course with shorter time between symptom onset to death, long duration to being symptom-free and to being discharged. Whereas Kyle et al. (49) report that the course of the disease in neonates is usually mild. Similarly, in their journal article, De Bernardo et al. (21) reported that COVID-19 in neonates showed a good prognosis, with severe complications and death being rare. Further, other studies reported that infection in the neonatal period runs a mild course and that the outcome is favourable (43, 30, 31, 53). Furthermore, the WHO Scientific brief of 8th February 2021 (108–111) stated that neonatal infection of SARS-CoV-2 usually presents with non-severe symptoms.

In order to be certain about vertical transmission a neonate should be tested more than once and a positive test should be confirmed with a molecular test, RT PCR. Because maternal IgG unlike maternal IgM crosses the placenta, IgG should not be used to diagnose infection in the neonate. Instead a positive IgM test in a neonate aged at least one week old should raise suspicion for neonatal infection. Positive SARS-CoV-2 test in a neonate under the age of one week is most likely representative of maternal infection. But negative IgM at age less than one week followed by positive test after age of one week represents active infection. Because the sensitivity and specificity of a positive IgM test is variable and less reliable than that of PCR, so positive serological test (IgM) should be followed by a molecular test (PCR). However, care should be taken in interpreting a positive RT-PCR test result because it may indicate at least one of the following possibilities; active infection, contamination with viral fragments during vaginal delivery or from the environment. Therefore, a positive PCR test should be repeated and also tested on normally sterile body fluids such as cerebrospinal fluid to further confirm the diagnosis (7, 9). To date, there has been no report in literature of detection of SARS-CoV-2 in amniotic fluid and breast milk (84). And de Medeiros et al. (22) further report that the absence of evidence to support the presence of the virus in the amniotic fluid, placenta, umbilical cord and breast milk of the expectant mothers points to the unlikelihood of vertical transmission of the infection.

Therefore, the risk of vertical or horizontal transmission of SARS-CoV-2 from the mother to the neonate is low (41, 99–107, 60, 114).

## 5. Precautions to take during breastfeeding

Generally all children, and more specifically neonates, infants and toddlers should be handled with extra care during this period of the pandemic so as to reduce the risk of transmission. More caution should be exercised if there is risk of transmission.

A woman with COVID-19 can and should be encouraged to breastfeed, as long as necessary precautions are taken, including (99–107, 61):

i. Wearing a face mask correctly and consistently.
ii. Washing hands with soap and water or use alcohol-based hand sanitizer.
iii. Routinely cleaning and disinfecting surfaces.

Adherence to the above listed precautions is important because enhanced hygiene is a proven measure of limiting the risk for transmission. Additionally, breast hygiene is to be practiced in order to reduce the risk of transmission.

## 6. Breastfeeding and vaccination

COVID-19 vaccine has emerged as one of the most effective methods of primary prevention of COVID-19 (38, 97). There are several brands of the COVID-19 vaccine available on the international market (32, 27). Vaccines typically work on the same principle; stimulating the immune response to weakend or inactivated parts of a particular antigen (32, 99–107). By so doing a vaccine stimulates the immune system to produce antibodies, similar to how it would if you were infected. After invading the cell, the agent is recognised as foreign material by the immune system which develops specific immune response against it. In the event of future exposure to the natural antigen, the body produces a more robust and quick immune response that would have otherwise not been produced (37, 18). In this way, therefore, vaccines prevent diseases caused by a large number of viruses, which could even have been fatal in the absence of vaccination as the body's immune system could not have mounted an adequate immune response.

Because lactating mothers were initially excluded from clinical trials much data regarding COVID-19 vaccines and breastfeeding are lacking. Because of the same reason, data on which to base decision making in this population is also not as available (34). But it is safe to vaccinate breastfeeding women (8) and/or their infants as the non-replicating vaccines which are now authorized for use do not reproduce inside host cells. Therefore several important societies advise that lactating women can be offered the vaccine as long as they are otherwise eligible, with the American College of Obstetricians and Gynecologists (ACOG) and United Nations Children's Fund (UNICEF) among others stating that there is no need to interrupt breastfeeding after vaccination (92, 12–14, 20). Therefore, lactating women may choose to be vaccinated. And there is no contraindication to the administration of this vaccine alongside other vaccines that are otherwise routinely administered in pregnancy (70). A prospective cohort study by Gray KJ et al. (34) enrolled 131 women of reproductive age who had been vaccinated. Of the 131 women, 84 (64%) were pregnant, 31 (24%) were lactating, and 16 (12%) were non-pregnant. The study reviewed that “COVID-19 mRNA vaccines” caused a “robust humoral immunity” with comparable results in all the subjects irrespective of their pregnancy status. Furthermore, the study reviewed that the immunity arising from vaccination was of more robustness than that which was naturally occurring.

There have been no differences in the side effects arising from vaccination observed in the pregnant and the non-pregnant populations; also there have been no safety concerns from the CDC v-safe post-administration surveillance data (12–14). Furthermore, more evidence is becoming available to support the safety of COVID vaccination in pregnancy (12–14, 94). Although eligible to be vaccinated against SARS-CoV-2, pregnant women should be encouraged to minimize contact with other people. This is in order for them to reduce their chances of exposure to persons with SARS-CoV-2 infection, and to reduce their chances of contracting the infection. Despite the ACOG's (70) encouraging vaccination of pregnant women in any trimester of pregnancy, precaution should also be taken if the vaccine is to be administered during the first trimester. This is because of the likelihood of vaccine reactogenicity of which fever is the most important because it can potentially raise the risk of congenital malformations such as neural tube defects (73).

Although the COVID vaccines are a pivotal component of the COVID response globally, there is currently no basis to offer them to neonates because they were not included in clinical trials. And because neonates have both innate and adaptive immune systems that are still developing, they have poor immunogenicity and are not likely to generate adequate immune response to the vaccine (77, 96). Therefore, women who are planning to get pregnant can be encouraged to get vaccinated so that they can generate a sufficient repertoire of immunoglobulins to pass to the foetus when they get pregnant and confer passive immunity to neonate (96, 50). But this should be done with the caution already discussed above; to avoid administration of the vaccine in the first trimester. Because of their being a higher risk of potentially more severe disease and mortality from COVID-19 infection during pregnancy and that immunity from primary vaccine can wear off, the ACOG recommends that pregnant women and those in the puerperal period (≤6 weeks after delivery) should receive a bilavent mRNA booster vaccination not before 2 months after receiving their first dose or monovalent booster have elapsed (70). Significant increase in neutralizing SARS-CoV-2 specific antibodies in breast milk has been detected following COVID-19 vaccination similar to the phenomenon that occurs as after infection. It has therefore been suggested that these antibodies are passed on to the infant during breastfeeding. This is because the same antibodies have been identified in the stools of the infants 6 months after vaccination despite decrease in antibody levels (80).

Despite the foregoing evidence regarding the benefits of vaccinating both the pregnant and breastfeeding women to both the women themselves and the infants, there is vaccine hesitancy particularly against the COVID-19 vaccine than the other vaccines. Therefore, there is need for the health care providers to be aware of this and work to dispel the false information regarding COVID-19 vaccines and promote vaccination against COVID-19 (67).

## 7. Evolution of COVID-19 guidelines over time

The COVID-19 guidelines in general and those particularly concerned with prevention of the disease in young children have undergone several changes over time. For example on 18th February 2020 the CDC (40) published the guidelines that recommended that infants born to COVID-19 positive mothers should be temporarily separated from their mothers until they were negative. During the period of temporary separation milk expressed from the mother's breast was to be fed to the infant by healthy caregivers. 2 months after that, on 4th April of the same year the CDC guidelines promoted keeping the mother and the new born infant together and to continue breastfeeding. But their update would on 20th May 2020 change back to discouraging keeping the COVID-19 positive mother and her new born infant together and breastfeeding. Finally, on the 3rd August 2020, their guidelines were again changed to encourage the practice of rooming in and breastfeeding while observing COVID prevention measures. The guidelines regarding isolation of COVID patients have also undergone changes. The WHO guideline published on 12th January 2020 (99–107) recommended that there was supposed to be evidence of clearance of the virus before a patient could be discharged from isolation. This was to be proven by clinical recovery and having negative RT-PCR results on two separate occasions taken not less than 24 h apart. But with new scientific evidence becoming available, the updated guidelines published on 27th May 2020 recommended that patients could be discharged from isolation if they are symptom-free for 10 days after the start of initial symptoms (for symptomatic patients) and 10 days after testing positive for SARS-CoV-2 for asymptomatic cases. The WHO (81, 99–107) has maintained the guidance given on 13th March 2020 that new born infants of mothers with suspected or confirmed COVID-19 should not be separated from their mothers. But that they should remain with their mothers, breastfed normally with direct skin-to-skin contact, kangaroo mother care and rooming in all through the day and night further encouraged. During the period that they remain COVID-19 positive, mothers are encouraged to adhere to wash hands frequently, wear a face mask and to routinely disinfect surfaces. Breastfeeding is strongly recommended given its known lifelong importance for maternal and child health.

## 8. Conclusion

It is more beneficial to breastfeed than not to even during the pandemic as it is unlikely that breast milk would be the vector for MTCT of COVID-19. Breast milk also offers the best innate immunity to the growing infant. Therefore, safe breastfeeding should be encouraged, with necessary precautions so that there is no transmission from the mother to the infant. Pregnant and lactating women can also choose to get vaccinated against the SARS-CoV-2. Vaccinating this group of women may even prove beneficial to their infants because passive immunity can be conferred on to the fetus *in utero* by trans-placental passage of the vaccine induced antibodies, and ex utero via breast milk (postpartum).

### 8.1. Weaknesses

The weaknesses of this review are that some issues such as the timing of vaccination of pregnant mothers that may be controversial have not been fully clarified. Additionally, most of the information is not readily available currently because the COVID-19 is a new disease. It would therefore be important for further research to study the possibility of vaccinating children, efficacy and safety of COVID-19 vaccines in children and the most appropriate timings for vaccinating pregnant and lactating mothers. It would also be important for the studies to be conducted with bigger sample sizes and in multiple sites.
